# High serum unconjugated bilirubin levels following successful treatment of antiepileptic drug-induced bullosa epidermolysis: a case report and review of the literature

**DOI:** 10.3389/fmed.2025.1662795

**Published:** 2025-10-02

**Authors:** Chen Cheng, Yangyang Sun, Bingfeng Zhang, Huaguo Xu, Jian Xu, Jiexin Zhang

**Affiliations:** ^1^Department of Laboratory Medicine, The First Affiliated Hospital with Nanjing Medical University, Nanjing, China; ^2^Branch of National Clinical Research Center for Laboratory Medicine, Nanjing, China

**Keywords:** lamotrigine, levetiracetam, bullosa epidermolysis, methylprednisolone, unconjugated bilirubin, creatine kinase, case report

## Abstract

Most of the classical antiepileptic drugs (AEDs) such as lamotrigine (LTG) and levetiracetam (LEV) are known to potentially cause skin lesions and liver injury. This paper reports an unusual phenomenon that serum levels of unconjugated bilirubin (IBIL) abnormally increased after the successful treatment of AED-induced bullosa epidermolysis, regardless of the presence of genetic mutations associated with hepatocellular bilirubin metabolism. A 14-year-old female, who was suffering from epilepsy, presented to our hospital with fever and red papules all over her body. Based on histopathological examination of the skin biopsy specimen and a medical history of LTG and LEV use, toxic epidermal necrolysis (TEN) was confirmed. After methylprednisolone treatment for 29 days, the skin lesions showed retreated but drug-induced liver injury (DILI) aggravated. Serum biochemical test results revealed elevated levels of serum total bilirubin, conjugated bilirubin, IBIL, gamma glutamyl transpeptidase, alkaline phosphatase, total cholesterol, and total bile acid. B-ultrasound examination indicated cholecystitis. The upper abdominal magnetic resonance cholangiopancreatography was performed four times on her and revealed progressive dilatation of the biliary ducts and cholestasis. A whole-exon sequencing of the liver biopsy specimen revealed a heterozygous 1249G>A mutation in the *ATP binding cassette subfamily C member 2* gene and a heterozygous 211G>A mutation in the *UDP glucuronosyltransferase family 1 member A1* gene. Hepatobiliary protection regimen was administered for a total of 71 days to reduce serum bilirubin level and to relieve symptoms. Nevertheless, the patient developed chronic DILI. The serum IBIL levels showed limited response to the regimen and instead increased to as high as 344.6 μmol/L. After taking phenobarbital, the IBIL level decreased only to 270.2 μmol/L. However, we documented a strong inverse correlation between serum creatine kinase (CK) and IBIL levels, which has not been discussed in other reported cases of AED-induced liver injury. This case emphasizes that the precise interpretation of serum hepatobiliary indexes is important for differentiating energy imbalance from organic integrity. A combination of serum CK and IBIL may serve as a useful laboratory tool for DILI stage assessment.

## Introduction

The development of hypersensitivity to lamotrigine (LTG) and levetiracetam (LEV), which often occurs within a month after the initiation of the antiepileptic drugs (AEDs), in Asian populations is a cause of concern (1, 2). The clinical symptoms are skin lesions [e.g., Stevens–Johnson syndrome and toxic epidermal necrolysis (TEN)] and drug-induced liver injury (DILI), including acute hepatitis, cholestasis, bile duct injury, and fulminant hepatic failure (3). We herein report a case of extraordinarily high levels of serum unconjugated bilirubin (IBIL) following successful treatment of TEN. We also conduct a comprehensive review of the relevant literature, aiming to highlight its significance in clinical practice.

## Case presentation

A 14-year-old female presented with a 39 °C fever and scattered tiny red papules on the bilateral upper limbs without itching on June 2nd, 2023. She received intravenous infusions of latamoxef and ibuprofen, but the papules spread all over her body, including her face, neck, trunk, and bilateral thighs, and exhibited local fusion. She was admitted to the Emergency Department of our hospital on June 8th, 2023. The patient reported a medical history of epilepsy for 8 months and had no remarkable personal or family history of similar illnesses, using the AEDs LTG (75 mg bid) and LEV (250 mg bid) half a month before the onset of the papules. The serum biochemical test results at admission indicated in Table 1. The hemoglobin concentration, mean corpuscular hemoglobin concentration, erythrocyte count, reticulocyte count, eosinophil count, and international normalized ratio were within normal limits. The patient presented with jaundice of the entire skin and sclera. Punctate blood crusts were visible all over her body. There were no liver palms or spider angiomas. The liver and spleen were not palpable beneath the costal margin, and no abdominal mass was detected. Murphy’s sign was negative. The initial diagnosis was drug dermatitis. Methylprednisolone (80 mg) and gamma globulin (20 g) were administered for 2 days to control the symptoms.

**Table 1:** Serum biochemical test results at the time of clinical presentation.

Analyte	Result	Reference interval
Alanine aminotransferase	525.2 U/L	<35 U/L
Aspartate aminotransferase	539.9 U/L	0–45 U/L
Gamma glutamyl transpeptidase	193 U/L	10–78 U/L
Alkaline phosphatase	299 U/L	38–126 U/L
Creatine kinase	58 U/L	25–190 U/L
Total bilirubin	30.0 μmol/L	6.0–22.0 μmol/L
Conjugated bilirubin	5.0 μmol/L	0–5.0 μmol/L
Unconjugated bilirubin	8.5 μmol/L	0–19.0 μmol/L
Total protein	76.2 g/L	63.0–82.0 g/L
Albumin	44.1 g/L	35.0–50.0 g/L
Creatinine	56.6 μmol/L	44.0–132.0 μmol/L
Urea	3.22 mmol/L	2.10–7.20 mmol/L
Glucose	5.59 mmol/L	3.6–6.2 mmol/L

The patient was transferred to the Dermatology Department on June 12th, 2023. Measurement of serum immunoglobulin (Ig) levels revealed that IgE was 789.16 KU/L (reference interval: 0–60 KU/L), IgG was 23.4 g/L (reference interval: 8.6–17.4 g/L), IgM was 0.996 g/L (reference interval: 0.5–2.8 g/L), and IgA was 1.49 g/L (reference interval: 1.0–4.2 g/L). Skin tissue biopsy results confirmed TEN. She immediately stopped LTG and LEV use and received methylprednisolone (80 mg), glutathione, vitamin C, calcium gluconate, and nutritional support for nine consecutive days. Serum alanine aminotransferase (ALT) level decreased to 92.8 U/L, and aspartate aminotransferase (AST) level was 236.7 U/L. Serum levels of total bilirubin (TBIL), conjugated bilirubin (DBIL), and IBIL gradually increased to 101.2 μmol/L, 77.5 μmol/L, and 23.7 μmol/L, respectively. B-ultrasound examination indicated cholecystitis. One dose of Yisaipu (a recombinant human tumor necrosis factor-*α* receptor II Fc fusion protein) was administered subcutaneously on June 21st and June 25th for drug eruption treatment. Upper abdominal magnetic resonance cholangiopancreatography (MRCP) on July 3^rd^ revealed no significant abnormalities in the biliary ducts or in the pancreatic ducts. During this period, methylprednisolone (60 mg) was constantly administered along with ursodeoxycholic acid, magnesium isoglycyrrhizinate, and adenylmethionine succinate for hepatobiliary protection. Notably, serum TBIL level reached 195.8 μmol/L on July 3rd. Serum levels of DBIL and IBIL were 126.6 μmol/L and 69.2 μmol/L, respectively. Since there were no new skin lesions, most plaques had retreated and healed, and cutaneous Ney’s sign was negative, the patient was transferred to the Infectious Disease Department on July 7th, 2023, for DILI.

**Figure 1:**
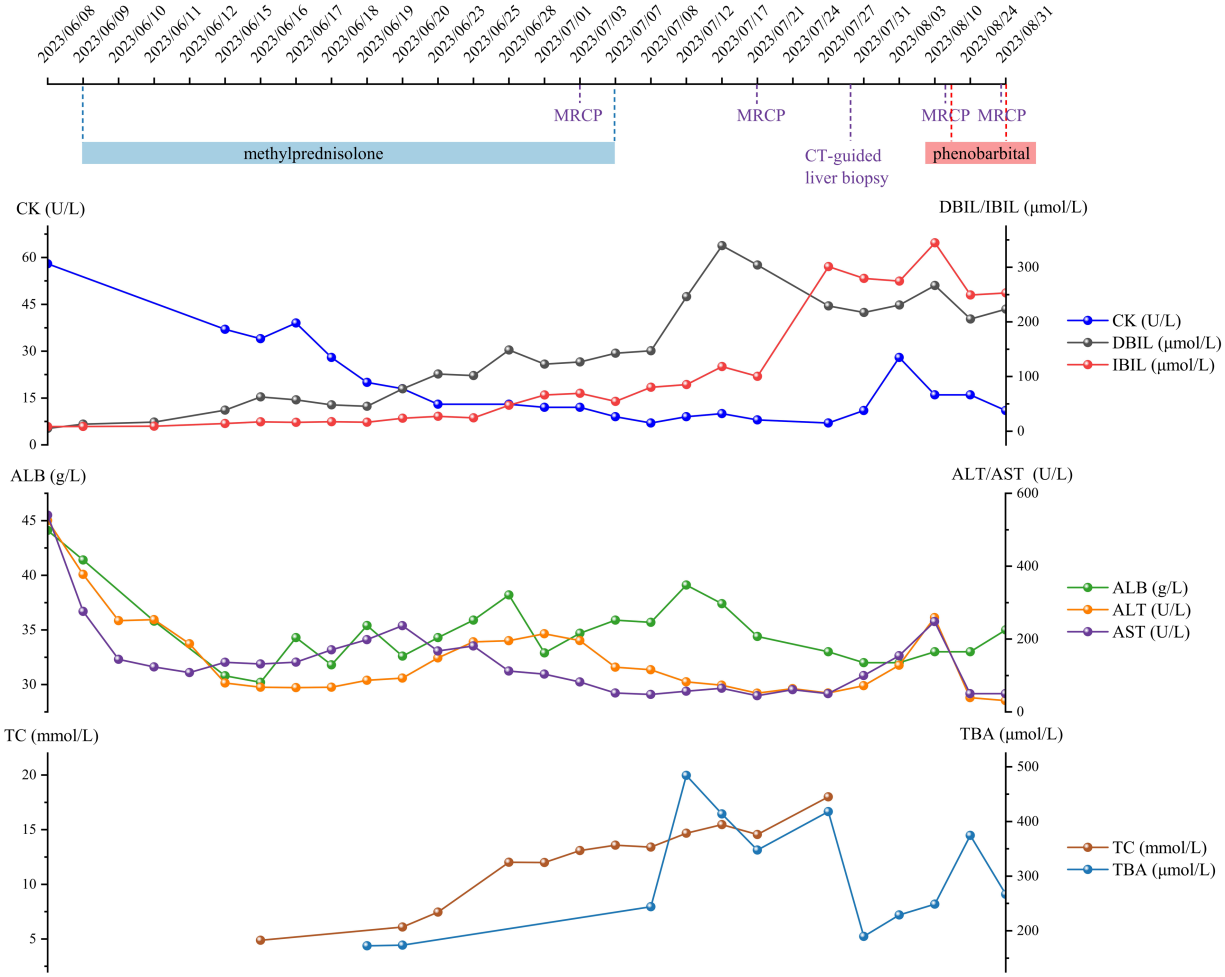
Clinical course and serological indicators related to liver function.

Figure 1 shows the serum levels of ALT, AST, DBIL, IBIL, creatine kinase (CK), albumin, total cholesterol, and total bile acid during hospitalization. The serological tests for hepatitis viruses, Epstein–Barr virus, cytomegalovirus, herpes simplex virus, and human immunodeficiency virus were negative. Indexes associated with primary biliary cirrhosis and autoimmune disease were all within the reference ranges. The stool presented white color, and the urine presented deep yellow color. Computed tomography-guided liver biopsy was performed on July 29th, 2023, and tissue samples were analyzed via whole-exon sequencing. The results suggested a heterozygous 1249G>A mutation in the ATP binding cassette subfamily C member 2 (*ABCC2*) gene and a heterozygous 211G>A mutation in the UDP glucuronosyltransferase family 1 member A1 (*UGT1A1*) gene. MRCP examinations were conducted on July 21st, 2023, August 15th, 2023, and August 29th, 2023 revealed string-of-bead-like dilatations of both the intrahepatic and extrahepatic biliary ducts accompanied by cholestasis. The final diagnosis was severe cholestatic hepatitis, secondary cholangitis, and biliary ducts dilatation. The hepatobiliary protection regimen consisting of ursodeoxycholic acid, magnesium isoglycyrrhizinate, adenylmethionine succinate, polyene phosphatidylcholine, cholestyramine, and glutathione was administered to reduce the serum bilirubin level since July 7th, 2023. Phenobarbital (30 mg qd) was added to the regimen since August 16th, 2023. The patient developed chronic DILI. The serum TBIL concentration was 476.2 μmol/L and serum IBIL was 252.9 μmol/L when she was discharged from our hospital on August 31st, 2023.

The main biochemical test results upon discharge are listed in Table 2. Approximately one and a half years later, the patient continues to experience fatigue and hyperbilirubinemia, with occasional episodes of abnormal hepatic enzyme levels as of this writing.

**Table 2:** Serum biochemical test results at the time of discharge.

Analyte	Result	Reference interval
Alanine aminotransferase	31 IU/L	7–40 IU/L
Aspartate aminotransferase	50 IU/L	13–35 IU/L
Gamma glutamyl transpeptidase	213 IU/L	7–45 IU/L
Alkaline phosphatase	460 IU/L	35–100 IU/L
Creatine kinase	11 IU/L	22–269 IU/L
Total bilirubin	476.2 μmol/L	4.7–24.0 μmol/L
Conjugated bilirubin	223.3 μmol/L	0–6.8 μmol/L
Unconjugated bilirubin	252.9 μmol/L	0–20.0 μmol/L
Total protein	50 g/L	60–83 g/L
Albumin	35 g/L	35–55 g/L
Creatinine	35 μmol/L	53–97 μmol/L
Urea	2.0 mmol/L	2.5–7.1 mmol/L
Total bile acid	266.8 μmol/L	1.0–10.0 μmol/L

## Discussion

Multidrug resistance protein 2 (MRP2, gene symbol *ABCC2*) is one of the principal hepatobiliary transporters in the intact liver. The nonsynonymous mutation 1249G>A in the *ABCC2* gene results in the replacement of the valine residue with an isoleucine residue at position 417 (V417I), which is located in intracellular loop 1 of transmembrane domain 1 of the MRP2 protein. Both *in vitro* and *in vivo* experiments confirmed that the expression level of the V417I mutant and its localization in the canalicular membrane were comparable to those of wild-type MRP2 in polarized hepatocytes. The V417I mutant has no effect on the serum DBIL level and is not associated with toxic or cholestatic hepatitis (4, 5).

Aberrant transcriptional and posttranscriptional regulation of MRP2 has been observed during obstructive cholestasis, and MRP3 in the basolateral membrane compensates for MRP2 deficiency in the livers of cholestasis patients (6). In this case, the patient exhibited high levels of serum ALT, AST, GGT, ALP, and TBIL on admission. Nevertheless, the serum DBIL concentration was 5 μmol/L. It gradually increased with the progression of biliary duct dilatation.

The patient also had a heterozygous mutation in *UGT1A1*, 211G>A (p.Gly71Arg, G71R), which is related to Gilbert’s syndrome and Crigler–Najjar syndrome. The protein encoded by the *UGT1A1* gene is a key enzyme involved in bilirubin biotransformation in the liver and selectively catalyzes IBIL glucuronidation to form DBIL (7). Although the 211G>A mutation is not one of the major genetic determinants of systemic bilirubin concentrations, the enzyme activity of a single copy of the G71R mutant is approximately 60% of that of the wild-type enzyme, and this mutant can cause unconjugated hyperbilirubinemia (8). Kimura et al. (9) reported four Asian acute leukemia patients who were heterozygous for the G71R mutant and had normal serum bilirubin concentrations at recruitment. Unconjugated hyperbilirubinemia (peak serum IBIL level of ~40 μmol/L) accompanied by an increase in the serum ALT level was reported during chemotherapeutic drug administration, and the serum bilirubin level returned to normal between the two therapeutic courses. Zhou et al. (10) recently described a Chinese family with concurrent *UGT1A1* mutations (including 211G>A) and *ABCC2* mutations (not including 1249G>A). The proband’s brother, who exhibited the same genetic defects as the proband, had a long-term history of medication use. When he was diagnosed with cirrhosis and primary hepatocellular carcinoma at 48 years of age, hyperbilirubinemia worsened from mild (IBIL level of 17.7 μmol/L, DBIL level of 45.8 μmol/L) to severe (IBIL level of 85.3 μmol/L, DBIL level of 286.1 μmol/L) within 2 years. The levels of both forms of bilirubin were higher than those in the proband at age 50 despite the absence of overt liver-related disease.

UGT1A1 has been implicated in a series of detoxification processes. Studies have confirmed that UGT1A1 plays a secondary role in the formation of the principal N-glucuronide metabolite of LTG in the liver. In patients with UGT1A1 variants, LTG clearance is decreased (11). Elimination failure and hepatocytic retention of LTG might accelerate liver damage. In this case, the patient presented with a mixed type of hepatocellular injury and cholecystitis. Her serum IBIL levels showed limited response to the liver-protective regimens and instead increased to as high as 344.6 μmol/L. After taking phenobarbital, the serum IBIL level decreased only to 270.2 μmol/L. Phenobarbital exerts its effects by activating the residual enzymatic activity of UGT1A1. Experimental phenobarbital treatment is often effective for Crigler–Najjar syndrome type II, with a reduction of approximately 25 to 30% in serum TBIL levels (12). In this case, the suboptimal therapeutic effect of phenobarbital in reducing serum IBIL levels indicated the impairment of hepatocyte transformation function.

Cholestasis impairs hepatic mitochondrial function and disrupts ATP formation. ATP-consuming biochemical reactions, including bilirubin export, cholesterol metabolism, and ureagenesis, are blocked by cholestasis which exacerbates hepatotoxicity. However, there was no obviously correlation between an increase in the serum IBIL level and abnormalities in other indexes in patients who experienced severe side effects from AEDs (13). Notably, the short-term increase amplitude of serum IBIL in this patient was apparently more marked than that in other reported cases of AED-induced DILI, regardless of the presence of genetic mutations (Figure 1). In addition, the abnormal increase in IBIL and cholesterol after the patient administered methylprednisolone suggests that excessive IBIL cannot be converted into DBIL and cholesterol cannot be converted into bile acids, indicating that the biotransformation function may be compromised.

CK is enriched in organelles with a high energy supply, such as hepatocellular mitochondria where CK catalyzes the reversible reaction between ATP and phosphocreatine. CK accelerates liver regeneration by enhancing ATP synthesis and utilization (14). However, the impact of low CK activity during DILI has not received much attention to date. Methylprednisolone is a synthetic corticosteroid that exerts antiallergic, anti-inflammatory, and immunosuppressive functions to treat drug eruption. In this case, methylprednisolone was the only drug that strongly suppressed CK activity (15). As shown in Figure 1, the opposite changes in serum IBIL and CK levels were indicative of a hypodynamic biotransformation reaction from IBIL to DBIL due to insufficient ATP participation. It is reasonable to assume that the combination of mitochondrial dysfunction and CK inefficiency comprehensively accelerated DILI progression to a chronic stage, as indicated by the serum IBIL concentration. Future research will focus on elucidating the relationship described above using on a large sample size.

## Conclusion

This report documents, for the first time, a case of rare unconjugated hyperbilirubinemia and a significant negative correlation between serum IBIL and CK that manifested following the successful treatment of AED-induced bullosa epidermolysis. During methylprednisolone treatment, the significance of low serum CK levels has been underestimated and overlooked, which can be associated with the severity of cholestatic hepatitis. The precise interpretation of serum hepatobiliary indexes is important for differentiating energy imbalance from organic integrity. If a next stage of DILI is emerging, more active therapeutic protocols should be considered to help the liver heal itself as soon as possible.

## Data Availability

The original contributions presented in the study are included in the article/supplementary material, further inquiries can be directed to the corresponding authors.
